# Orthopedic management of myelomeningocele with a multidisciplinary approach: a systematic review of the literature

**DOI:** 10.1186/s13018-021-02643-8

**Published:** 2021-08-13

**Authors:** Parnian Shobeiri, Ana Presedo, Amirali Karimi, Sara Momtazmanesh, Fardis Vosoughi, Mohammad Hossein Nabian

**Affiliations:** 1 School of Medicine, Tehran University of Medical Sciences (TUMS), Tehran, Iran; 2 Universal Scientific Education and Research Network (USERN), Tehran, Iran; 3 Non–Communicable Diseases Research Center, Endocrinology and Metabolism Population Sciences Institute, Tehran University of Medical Sciences, Tehran, Iran; 4 Department of Pediatric Orthopedics, Hôpital Robert Debre, Paris, France; 5 Department of Orthopedic and trauma surgery, Shariati Hospital and School of Medicine, Tehran University of Medical Sciences (TUMS), Tehran, Iran; 6 Center of Orthopedic Trans-Disciplinary Applied Research (COTAR), Tehran University of Medical Sciences (TUMS), Tehran, Iran

**Keywords:** Interdisciplinary, Communication, Meningomyelocele, Orthopedics, Spinal dysraphism

## Abstract

**Background:**

Myelomeningocele (MMC) is the most common and severe form of spina bifida and imposes a significant burden on patients and the healthcare system. Recently, the multidisciplinary management of MMC has become popular. Herein, we aimed to review the orthopedic management, outcomes, and complications of the of patients with MMC eyeing a multidisciplinary approach.

**Methods:**

We searched PubMed and EMBASE to find relevant studies published before August 2020. All studies that included clinical management of MMC patients and published earlier than 2000 were considered for review on the condition that they reported at least one orthopedic intervention and the rate of complications. We excluded review articles, case reports, case series, letters, commentaries, editorials, and conference abstracts. The primary and secondary goals of our review were to report the outcomes and complication rates of multidisciplinary management for MMC patients.

**Results:**

Twenty-six studies included data for the management of 229,791 patients with MMC and were selected. Sixteen studies reported multidisciplinary management in addition to orthopedic management. From those, 11 (42.31%) included urologic management, 13 (50%) neurosurgical management, 11 (42.31%) neurologic management, and 5 (19.23%) gastrointestinal management. All studies included postnatal operations and related management. No randomized clinical trial was found in our search.

**Conclusion:**

Orthopedic approaches play a key role in MMC management by alleviating spinal deformities, particularly scoliosis, and hip, foot, and ankle complications. However, the most appropriate management, whether surgical or non-surgical, may vary for different patients, given disease severity and the age of patients.

**Graphical abstract:**

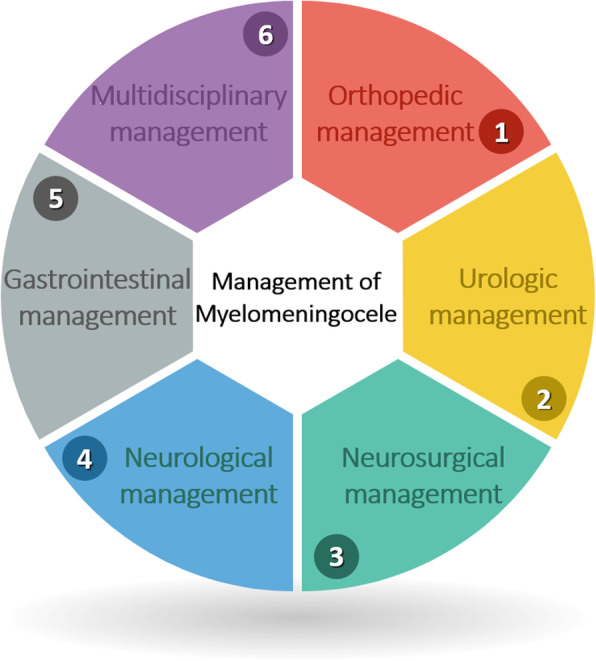

## Background

Spina bifida (SB) represents a congenital cause of disability characterized by an opening in the vertebral column [1]. In 2004, this condition held the sixth place among various congenital birth defects resulting in hospital admission in the USA [2]. According to the registry of European Surveillance of Congenital Anomalies (EUROCAT) in 2003–2007, the prevalence of SB was 0.51 per 1000 pregnancies, which makes it the most prevalent neural tube defect (NTD) [3]. Myelomeningocele (MMC), also known as open SB or SB aperta, is the most common and severe form of SB, inflicting a tremendous burden on patients and the healthcare system [4]. The responsible mechanism for this condition is the failure of neural tube closure during embryonic development, which results in a protruded spinal cord and meninges. This process leaves the underlying neural tissue vulnerable to damage [1, 5]. MMC can be associated with other congenital disorders such as hydrocephalus and Chiari II malformation [5–8].

Orthopedic consequences may arise from MMC involving various parts of the body, including feet, spine, and hip [9, 10]. Several orthopedic methods are used to manage these disabilities with variable success [11–14], and we will give a brief introduction regarding them in this “Background” section. Controversies regarding these methods are not addressed adequately and requires research and attention.

Foot consequences affect 80–95% of the patients and can present by clubfoot, valgus, equinus, calcaneus/calcaneovalgus, talus, etc. [15, 16]. The Ponseti method of clubfoot, soft-tissue release, different types of osteotomy, and hemiepiphysiodesis for valgus are among the methods used to correct these abnormalities [16]. Skin complications, such as wound infection and pressure sores, osteomyelitis, delayed-union or non-union of the bones, loss of correction, avascular necrosis, and recurrence are among the complications that vary largely based on the condition and utilized technique [16–19].

Spinal surgery mostly involves correcting kyphosis, scoliosis, or lordosis. These consequences affect patients’ appearance, ambulation, and quality of life by imposing consequences such as back pain and less social and personal acceptance [20, 21]. Some of the surgical and non-surgical management include spinal fusion, spinal orthoses, and sitting supports [13, 22]. These treatments may cause several complications, including decreased ambulation, pressure sores and other infections, implant problems, neurologic consequences, and interference with other care such as self-catheterization [23–25].

Muscle paralysis and imbalance may result in hip deformities, such as dislocation, subluxation, or contracture [26]. Surgical reduction may benefit a small population of the patients based on their level of MMC, while preserving muscle strength and maintaining the pelvic level and motion may assist many others [27, 28]. Surgical adverse effects include pathologic fractures and loss of ambulation [28, 29].

Various other sequels may occur involving other organs depending on the level of MMC, including motor and sensory deficits, urological problems, and bowel incontinence [30–34]. Such complications may cause ambulation difficulties, diminished quality of life, and restriction of the patients’ ability to attend school [34–37]. Managing these potentially unfavorable consequences require a multidisciplinary approach and highly specialized care.

In this systematic review, we summarize and compare the foremost treatment approaches proposed by researchers and specialists in the field of orthopedics, eyeing a multidisciplinary approach towards other fields.

## Material and methods

### Search strategy

This systematic review was conducted based on the guidelines of the Preferred Reporting Items for Systematic Reviews and Meta-Analyses (PRISMA) [38]. This review was registered (#CRD42021225916) in the International Prospective Register of Systematic Reviews (PROSPERO) and the review protocol can be accessed [39].

We included all studies that evaluated MMC patients’ clinical management if they reported: (1) at least one type of orthopedic intervention, (2) the rates of complications, and (3) published earlier than 2000. We excluded review articles, case reports, case series, letters, commentaries, editorials, and conference abstracts.

### Literature search and information sources

**Fig. 1:**
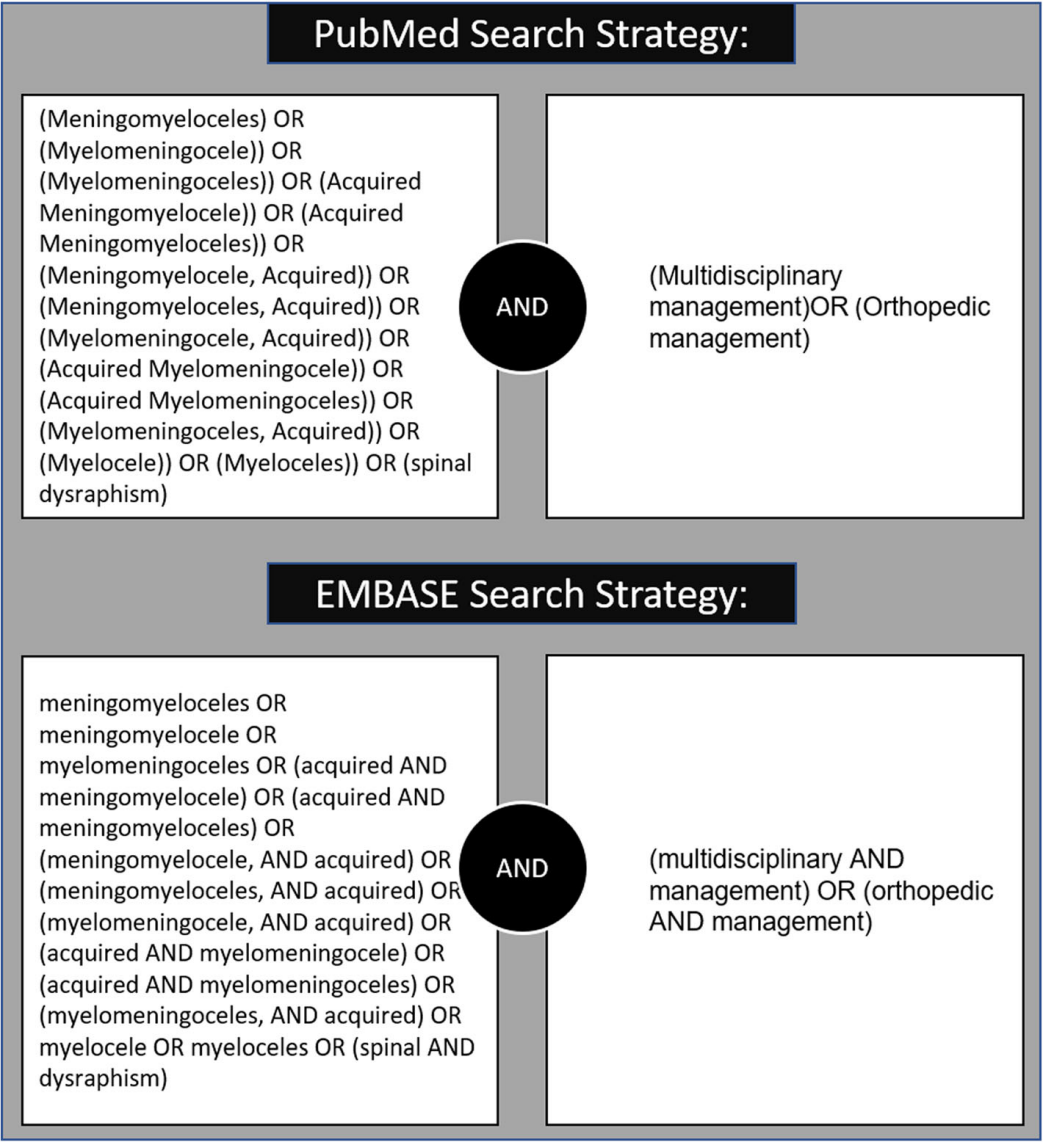
Search strategy

Before conducting this review, we searched PROSPERO and found no systematic review with a similar topic. To identify relevant publications, we searched PubMed and EMBASE databases in August 2020. Our search protocol was designed based on MeSH (MEDLINE) keywords and Emtree (EMBASE). The main search terms included MMC, spinal dysraphism, spina bifida, multidisciplinary management, and orthopedic management. The search was performed without time, country, or language limitations. The comprehensive search strategy is illustrated (Fig. 1).

### Study selection and data collection

**Fig. 2:**
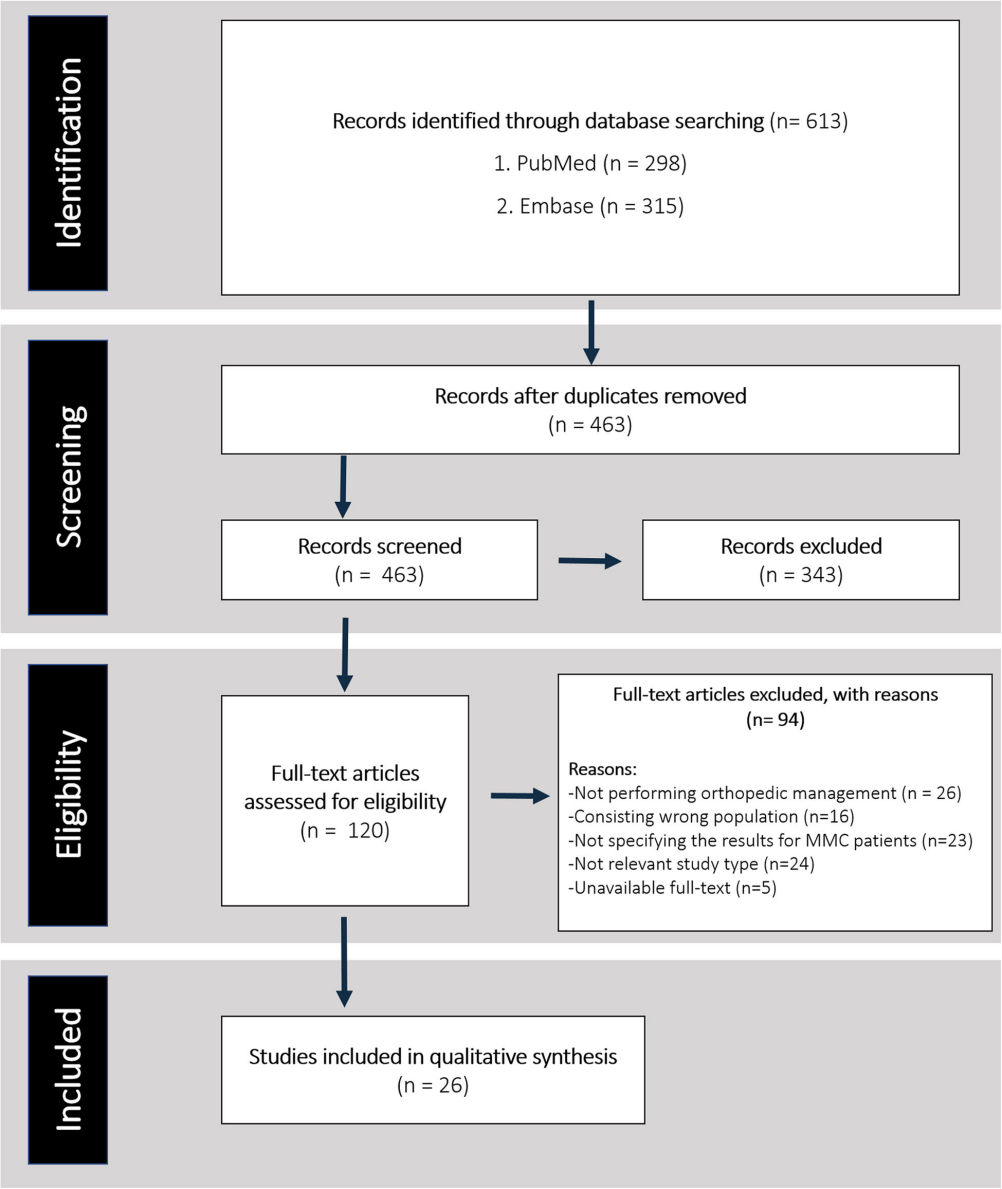
Study selection process according to the Preferred Reporting Items for Systematic Reviews and Meta-Analyses (PRISMA) guideline

All resulting records were imported to EndNote (version X20, Thomson Reuters, Toronto, Ontario, Canada), and duplicates were removed. Two review authors (P.S. and A.K.) independently screened all titles and abstracts. Then, full texts of all potentially relevant publications were assessed for eligibility. To find additional records, we reviewed the reference lists of the included papers. Any discrepancies between the two reviewers were solved out via discussion and consensus. If the disagreement persisted, a third reviewer (S.M.) was consulted. The PRISMA study selection diagram is illustrated to show the study selection process (Fig. 2). Two authors (P.S. and A.K.) independently extracted data from the included studies. We designed a data extraction form which included the following items: (1) general information (name of the first author, publication year, location of study, and study title), (2) patients’ characteristics (number of patients and controls, mean age, sex ratio, and location of MMC), (3) management methods, (4) rates of complications, and (5) outcome of interventions.

### Outcome measures

In this review, the primary outcome was the result of orthopedic management for MMC patients. Besides, we summarized other accompanied managements (e.g., urologic, neurosurgical and neurological, gastrointestinal) to reach the purpose of conducting a multidisciplinary review. Additionally, we investigated the complications of these interventions.

### Quality assessment

The quality of the included studies was assessed using the Newcastle–Ottawa Scale (NOS) [40]. Based on this scale, each study can receive a score of 0–9 based on the quality of the sample selection (max 4 scores), comparability of cases and controls (max 2 scores), and assessment of the exposure/outcome (max 3 scores). In this quality assessment tool, a lower score indicates a greater risk of bias.

## Results

### Study selection

The search yielded 613 results (Fig. 2). After removing 150 duplicated records, our screening method led to the exclusion of 343 articles. We assessed 120 full texts for eligibility. Finally, a total of 26 studies were included [9–14, 20, 21, 26, 41–57].

### Study characteristics

✓From the 26 articles included, 19 were retrospective cohorts [9–14, 20, 21, 41–45, 49, 51, 54–57], 4 were prospective cohorts [26, 46, 52, 53], 2 were retrospective cross-sectional studies [47, 48], and 1 was a case-control study [50]. We systematically reviewed clinical management of 229,791 patients with MMC whose treatment involved at least one kind of orthopedic intervention. Sixteen studies provided other types of managements, besides orthopedic approaches, which were as follows: 11 (42.31%) urologic management [9–11, 44, 45, 50, 51, 53, 55–57], 13 (50%) neurosurgical management [9–11, 20, 43–45, 49, 50, 53, 55–57], 11 (42.31%) neurologic management [9–11, 21, 44, 45, 49, 53, 55–57], and 5 (19.23%) gastrointestinal management [9, 11, 55–57]. Moreover, all the studies included described postnatal operations and management. Table 1 presents the characteristics of the selected studies.

**Table 1:** Characteristics of the included studies

Study	Study type	Sample	Age, gender, follow-up	Management					
				Orthopedics	Urology	Neurosurgery	Neurology	Gastrointestinal	Multidisciplinary
Torosian 2000 [42], USA	Retrospective cohort	*- N* = 27 (38 feet) *-* 38 feet in 27 patients,17 right and 21 left *-* Thoracic: 1 (4%) *-* Low lumbar: 18 (63%) *-* Sacral: 10 (33%)	*-* Mean age at surgery: 10.88 ± 2.50 *-* Gender: 16 (59.2%) males *-* Mean FU: > 5 years	✓					
Sponseller 2000 [41], USA	Retrospective cohort	*- N* = 48 *-* Children and adolescents who underwent Scoliosis surgery from 1986 to 1996	*-* Mean age at surgery: Infected 14.1 years, uninfected matched group 14 years *-* Note: CP patients also included *-* Gender: N/A	✓					
Frischhut 2000 [12], Austria	Retrospective cohort	*- N* = primarily 107 (final 91 patients and 182 feet) *-* Thoracic: 51 (28%) *-* Lumbar: 73 (40%) *-* Sacral: 58 (32%) *-* Patients with SB born before 1982	*-* Patients older than 15 years old *-* Gender: N/A	✓					✓
Parsch 2001 [13], Germany	Retrospective cohort	*- N* = 54 *-* Patients with surgical management for paralytic scoliosis between 1984 and 1996	*-* Mean age at surgery: 13.24 ± 2.09 years *-* Gender: 45/75 males (initial population before exclusion) *-* Mean FU: 3.3 years	✓	✓				
Crawford 2003 [20], USA	Retrospective cohort	*- N* = 11 *-* Neonates with MM who had a kyphectomy in conjunction with dural sac closure between 1980 and 2000	*-* Mean gestational age: 38 weeks *-* Mean age at surgery: 36 h *-* Gender: 9 (81.8%) males *-* Mean FU: 7 years and 4 months (range 44–174 months)	✓		✓			✓
Ozerdemoglu 2003 [43], USA	Retrospective cohort	*- N* = 105 (26 patients with scoliosis and MMC) *-* Patients with scoliosis and syringomyelia treated between 1941 and 1998	*-* Age: N/A *-* Gender: 47% males *-* Mean FU: 12.8 ± 9.6 years (range: 2–45.2 years)	✓		✓			✓
Ulsenheimer 2004 [44], Brazil	Retrospective cohort	*- N* = 31 *-* MMC neonates born between 1990 and 2000	*-* Median age at MMC correction: 4 days (range 1–44 days) *-* Mean gestational age: 39.5 ± 1 week *-* Gender: 16/31 (51.6%) males *-* Median FU: 24 months (range 6–68 months)	✓	✓	✓	✓		✓
Erol 2005 [26], Turkey	Prospective cohort	*- N* = 26 (28 hips) *-* Patients underwent surgery for hip instability between 1998 and 2001 *-* Patients previously underwent various surgical interventions other than orthopedic procedures, mainly urological and neurosurgical interventions (mean number of previous operations 2; range 1–4)	*-* Mean age at surgery: 4.5 years (range 3–6 years) *-* Gender: 16/26 males *-* Mean FU: 38 months (range 30–48 months)	✓					
Lemelle 2006 [45], France	Retrospective cohort	- *N* = 769 (421 were finally included) - Patients with at least 10 years of age who underwent surgery between 2003 and 2004 - All presented with MMC at the neonatal period	- Mean age: 22.1 years (range 10–47.5 years) - Gender: 191/421 (45%) males - Medically treated for urinary incontinence (*n* = 191) o Age: 21.7 ± 7.9 o Gender: 104/191 (54.4%) males o FU: N/A - Surgically treated for urinary incontinence (*n* = 230) o Age: 23.0 ± 7.6 o Gender: 126/230 (54.7%) males o Mean FU after initial surgery: 9.25 years	✓	✓	✓	✓		✓
Gerlach 2009 [46], USA	Prospective cohort	- *N* = 36 - 18 patients (28 feet) with MMC and clubfoot between 2001 and 2006 - 20 patients (35 feet) with idiopathic clubfoot prospectively gathered - Both groups managed with the Ponseti method	- Mean age at first cast for MMC patients: 12.4 weeks (range 1.2–25.9 weeks) - Gender: 9/16 males in MMC group - Mean FU for MMC patients:33.8 months (range 25.0–42.7 months)	✓					
Huang 2010 [49], China	Retrospective cohort	- *N* = 10 - Cervical MMC patients	- Median age at management: 3 months (range 9 days–8 years) - Gender: 6 (60%) males	✓		✓	✓		✓
Akbar 2010 [48], Germany	Retrospective cross-sectional	- *N* = 862 - Patients with MMC - 92 (10.7%) patients suffered from fractures	- Mean age: 10.2 ± 7.0 years - Gender: 391 (45.4%) males	✓					
Akbar 2010 [47], Germany	Retrospective cross-sectional	- *N* = 32 - MMC patients with Lumbar Kyphosis operated between 1994 and 2008	- Mean age at surgery: 7.3 ± 3.4 years - Gender: 13/32 (40.6%) males - Mean FU: 7.6 years (range 1.5–14.3 years)	✓					
Yildirim 2015 [14], Turkey	Retrospective cohort	- *N* = 13 - Patients with hip subluxation or dislocation secondary to lower lumbar MMC, surgically treated between 2004 and 2013	- Mean age at surgery: 6 years (range 21 months–12 years) - Gender: 8/13 (61.5%) males - Median FU: o Muscle transfer group: 36 months (range 14–122 months) o Non-muscle transfer group: 40 months (range 19–66 months)	✓					
Wang 2015 [50], USA	Case-control	- *N* = 226,709 cases and 888,774 controls - Nationwide Emergency Department Sample (NEDS) data between 2006 and 2010	- Mean age: 28.23 ± 0.58 years cases and 28.39 ± 0.13 years controls - Gender: 98,365 (43%) males in case group and 386,181 (43%) males in control group - FU: N/A	✓	✓	✓	✓	✓	✓
Januschek 2016 [53], Germany	Prospective cohort	- *N* = 48 - Meningocele:1 case - MMC: 29 cases - Myeloschisis:18 cases	- Mean gestational age at birth: 38 weeks - Gender: 25 (52%) males - FU: 9 weeks to 8 years	✓	✓	✓	✓		✓
Altiok 2016 [51], USA	Retrospective cohort	- *N* = 65 patients (MMC: 43 and Lipomeningocele: 22) - Thoracic and upper levels: 12 - L3–L4: 42 - Sacral: 11	- Mean age o Tethered cord release: 6.2 years o Definitive spine surgery: 11.6 years - Gender (TCR): 9 (43%) males - Mean FU: 3.8 years	✓	✓				✓
El-fadl 2016 [52], Egypt	Prospective cohort	- *N* = 27 (54 feet) - MMC patients with clubfoot (Talipes equinovarus)	- Mean age at first cast: 5.9 weeks (range: 3–8 weeks) - Gender: 9/24 (37.5%) males - Mean FU: 27 months (range 24–34)	✓					
Matar 2017 [54], UK	Retrospective cohort	- *N* = 11 (18 feet) - 18 MMC-associated clubfoot (7 bilateral deformities)	- Mean age: 4.7 weeks (range 2–8 weeks) - Gender: 4 (36.3%) males - Mean FU: 4.5 years (range 3–9)	✓					
Ryabykh 2018 [21], Russia	Retrospective cohort	- *N* = 20 - Patients with MMC-related spinal deformity	- Mean age at treatment: 6.3 years - Gender: 12 (60%) males - Mean FU: 34.5 months	✓			✓		✓
Spazzapan 2018 [56], Slovenia	Retrospective cohort	- *N* = 20 - Patients with MMC treated between 2007 and 2017	- All patients were operated within the first 48 h of life - Gender: 9/20 (45%) males - Mean follow-up: 7.7 years	✓	✓	✓	✓	✓	✓
North 2018 [55], Canada	Retrospective two-time cohorts	- *N* = 101 (C1) + 51 (C2) - Two cohorts: C1) born with MMC between 1971 and 1981 (data available from previously published work)**;** C2) born with MMC between 1996 and 2006 - Delivery (C2): 51% caesarean section, 49% spontaneous vaginal delivery	- Mean gestational age: 37.8 weeks C1 - Mean age at last FU (83 patients): 13.4 years (range 8.6–19.6 years) - Gender: 56/101 (55%) females C2 - Mean age at last FU (46 patients): 15.7 years (range 10.5–20.3 years) - Gender: 18/46 (39%) females	✓	✓	✓	✓	✓	✓
Beuriat 2018 [9], France	Retrospective cohort	- *N* = 46 - MMC children - Sacral or lower lumbar (≤ L4): 27 - Higher lumbar (L1-L3): 5 - Thoracic: 14 - Delivery: 20 cesarean sections and 26 vaginal delivery	- Mean gestational age at birth: 38 weeks and 6 days (range 33 weeks and 3 days–41 weeks) - Gender: 24/46 (52.2%) females - Mean duration of FU: 8.1 ± 4.6 years	✓	✓	✓	✓	✓	✓
Goldstein 2019 [10], USA	Multidisciplinary, retrospective cohort	- *N* = 208 - Patients undergoing scoliosis corrective surgery - Prophylactic untethering: 32 concomitant untethering, 21 untethering within 3 months prior, 155 without untethering	- Mean age at treatment: 9.4 years (range: 1–17 years) - Gender: 99 (47.6%) males - FU: 90 days	✓	✓	✓	✓		✓
Sileo 2019 [57], UK	Retrospective cohort	- *N* = 241 - Prenatally diagnosed isolated spina bifida - 67 underwent postnatal surgery	Maternal parameters: - Mean maternal age: 30.1 ± 5.9 years - Mean gestational age at diagnosis: 20.8 ± 1.9 weeks - Mean gestational age at birth: 38 ± 2.0 weeks Child parameters - Age at surgery: first few days - Mean age of children at the time of study: 6 years (range 0–18 years) - Mean duration of FU: 8.1 ± 4.1 years	✓	✓	✓	✓	✓	✓
Beuriat 2019 [11], France	Retrospective Cohort	- *N* = 29 - MMC with an anatomical level of L4 and below - 12 sacral - 17 lumbar	- Age range at surgery: 0–97 days - Mean age at delivery: o Cesarean section: 38 weeks and 2 days (range 37 weeks and 6 days–39 weeks) o Vaginal delivery: 39 weeks and 2 days (range 37 week and 7 days–41 weeks) - Gender: 13 (44.8%) females - Mean FU: 9.1 ± 4.9 year	✓	✓	✓	✓	✓	✓

*FU* follow-up, *N/A* not available

### Quality assessment

We found no randomized clinical trial (RCT) regarding this topic. Therefore, the risk of bias was assessed with the NOS for non-randomized studies (Table 2). We assessed the quality of 26 studies with a total of 234 items for the risk of bias.

**Table 2:** Newcastle-Ottawa Scale (NOS) risk of bias assessment of the included studies

Author, year	Selection (0–4)	Comparability (0–2)	Exposure/outcome (0–3)	Total score (0–9)
Torosian 2000 [42]	3	1	2	**6**
Sponseller 2000 [41]	2	2	3	**7**
Frischhut 2000 [12]	3	0	3	**6**
Parsch 2001 [13]	3	0	2	**5**
Crawford 2003 [20]	3	0	3	**6**
Ozerdemoglu 2003 [43]	4	0	3	**7**
Ulsenheimer 2004 [44]	3	0	3	**6**
Erol 2005 [26]	3	0	3	**6**
Lemelle 2006 [45]	4	0	3	**7**
Gerlach 2009 [46]	4	2	3	**9**
Huang 2010 [49]	3	0	3	**6**
Akbar 2010 [48]	4	0	3	**7**
Akbar 2010 [47]	3	0	3	**6**
Yildirim 2015 [14]	4	1	3	**8**
Wang 2015 [50]	4	2	3	**9**
Januschek 2016 [53]	3	0	2	**5**
Altiok 2016 [51]	3	0	3	**6**
El-Fadl 2016 [52]	3	0	3	**6**
Matar 2017 [54]	3	0	3	**6**
Ryabykh 2018 [21]	3	0	3	**6**
Spazzapan 2018 [56]	3	0	3	**6**
North 2018 [55]	4	0	3	**7**
Beuriat 2018 [9]	3	0	3	**6**
Goldstein 2019 [10]	4	1	3	**8**
Sileo 2019 [57]	3	0	3	**6**
Beuriat 2019 [11]	4	0	3	**7**

All studies received 3 or 4 points in the selection domain, except one study performed by Sponseller et al. [41]. The comparability was not adequately addressed in the majority of articles. Except for three studies [13, 42, 53], the exposure/outcome assessment was fully reported. Only two studies by Gerlach et al. and Wang et al. received a full score [46, 50]. The lowest score was 5, which was attributed to Parsch et al. and Januschek et al. [13, 53].

### Orthopedic management

Patients often need diverse management approaches based on the affected part of the body, including the spine, foot, and hip [58].

#### Foot

From the 26 studies included, five studies included interventions to correct foot deformities [12, 42, 46, 52, 54]. Four studies deal with clubfoot management [12, 46, 52, 54], two studies addressed the correction to valgus deformity [12, 42], and one study treated equinus deformity [12].

##### Clubfoot

Frischhut et al. [12] studied 91 patients with SB (182 feet). Clubfoot deformity was predominantly diagnosed in children with lumbar lesions (73 out of 182 feet). From the 37 feet corrected with surgery (via Cincinnati incision) before the age of 2 years, the recurrence of clubfoot deformity was observed in 29 feet by the age of 2, and 18 patients required a revision surgery by the age of 10. From the 73 cases associated with a lumbar lesion, bracing was used in 70 cases before the age of 2 and in 52 cases before the age of 10. At the last follow-up visit, at 24.5 years, only 2 cases needed revision surgery [12].

Gerlach et al., El-Fadl et al., and Matar et al. [46, 52, 54] used the Ponseti method to treat clubfoot deformity in a total of 74 patients. Matar et al. [54] defined the outcomes after treatment of clubfoot deformity: (1) achieving plantigrade pain-free feet, and (2) the need for surgical intervention. They followed 18 feet with an average Pirani score of 5.5 (range 3.5–6.0) and performed Ponseti casts and tendo-achilles tenotomy surgery in 17 (94.4%) feet. Fifteen feet (83.3%) were functional and pain-free at the final follow-up [54], 5 of which showed recurrence. Two other studies by Gerlach et al. and El-Fadl et al. [46, 52] used the Diméglio scoring system to assess the clubfeet. Based on four minor and four major criteria, this scoring system differentiates four groups of clubfoot: (I) benign (0–5; > 90% reducible), (II) moderate (5–10; > reducible but partly resistant), (III) severe (10–15; resistant but partly reducible), and (IV) very severe (15–20; almost irreducible) [59]. In the study of Gerlach et al., the mean overall Diméglio grade after treatment was 3.3 (range 3.0–3.6) in the MMC group. Out of 28 feet, 24 (86%) required percutaneous achilles tenotomy after the initial treatment, and 19 (68%) feet showed recurrence [46]. El-Fadl et al. reported that the mean Diméglio score improved significantly from 15.25 before the management to 7.42 at the last follow-up visit after the management (*P* < 0.001) [52].

##### Valgus

A valgus foot tends to be observed in mid and lower lumbar MMC, and hindfoot surgery is usually performed to correct this deformity [60]. Frischhut et al. reported 40 feet with calcaneo-valgus deformity (14 with L3–L4 lesions and 26 with L5–sacral lesions) at 10-year follow-up despite adequate treatment. Interestingly, this value increased to 48 (16 with L3–L4 lesions and 32 with L5–sacral lesions) at the last follow-up (24.5 years old on average) [12]. In the study of Torosian et al., postoperative outcomes showed that interventions had corrected the hindfoot alignment to approximately 5° of valgus. Also, the hindfoot alignment of feet varied from 0° to 10° [42].

##### Equinus and calcaneus

As reported by Frischhut et al., the equinus deformity was present in 37 feet at the age of 2 years (33 with T12–L2, 2 with L3–L4, and 2 with L5–sacral lesions) [12]. At 10-year follow-up, 41 feet showed equinus deformity (21 with T12–L2, 2 with L3–L4, and 18 with L5–sacral lesions), which was mainly related to an increased occurrence in patients with sacral lesions. The prevalence of calcaneus deformity decreased from 31 cases at 2 years of age (4 with T12–L2, 3 with L3–L4, and 24 with L5–sacral lesions) to 19 cases at 10 years (9 with T12–L2, 8 with L3–L4, and 2 with L5–sacral lesions). At the last follow-up visit (24.5 years on average), in patients with L5–sacral lesions, the calcaneus deformity had increased again up to 16 cases [12].

#### Spine

Ten out of the 26 included studies included corrective operations of the spine related to scoliosis in 5 studies [10, 13, 21, 43, 51], kyphosis in 3 studies [20, 21, 47], and lumbar lordosis in 2 studies [20, 21].

##### Scoliosis

Parsch et al. reported three surgical techniques: (I) posterior fusion (Cotrel-Dubousset instrumentation or Spine-Fix system) (*n* = 20), (II) anterior osteodiscectomy + posterior fusion (*n* = 12), and (III) anterior ventral derotational spondylodesis (VDS) + posterior fusion (*n* = 22) [13]. They measured the postoperative Cobb angle (median-IQR) for each group: (I) 35° (23°–49°), (II) 52° (37°–65°), and (III) 38° (30°–40°). The median-IQR for the loss of correction of scoliosis in each group was (I) 9° (− 2°–52°), (II) 10° (1°–52°), and (III) 7° (0°–26°). They reported that VDS combined with posterior fusion has resulted in a better midterm correction of scoliosis compared with posterior fusion alone (*P* = 0.02). Also, thoracic level paralysis had a more negative impact on the relative loss of correction compared with the lumbar level paralysis. They recommended the two-stage procedure in patients with a thoracic level of paralysis to reduce the risk of hardware complications and loss of correction [13].

Ozerdemoglu et al. indicated nonoperative treatment (passive observation and/or bracing) in 18 MMC patients and reported a curve progression of 3.4° per year [43]. Altiok et al. performed tethered cord release surgery (TCR) before scoliosis surgery. The average Cobb angle improved from 68.4° ± 21.8° before TCR to 44.5° ± 16.6° after corrective spine surgery [51]. Ryabykh et al. reviewed the outcome of instrumented fusion by pedicle screw-rod constructs in 20 patients. The preoperative mean Cobb angle for scoliosis was 36.7° (median: 36°, range 11°–77°) and the mean Cobb angle scoliosis correction was 25° (median 23°, range 1°–62°) [21]. Goldstein et al. used prophylactic spinal cord untethering within 3 months prior (*N* = 21) or concomitant (*N* = 32) with the corrective scoliosis surgery in MMC patients. They found an increased risk of complications, including surgical site infection (adjusted RR = 2.65, *p* = 0.02), rate of return to operation room (adjusted RR = 2.17, *p* = 0.045), the need for blood transfusion (*p* = 0.04), and increased mean length of stay (*p* < 0.0001). Moreover, they stated no increased risk of neurological injury without prophylactic untethering before scoliosis surgery in MMC patients [10].

##### Kyphosis

Crawford et al. investigated the effect of kyphectomy in combination with dural sac closure on 11 neonates [20]. They reported an average preoperative kyphotic angle of 67°, an average initial correction of 77°, and an average loss of correction of 55° at follow-up. Of the nine patients who were available for long-term radiographic follow-up (7 years and 4 months on average), four patients had kyphosis less than 67° (range 4°–67°), and three had kyphosis greater than 100° (range 100°–103°). Although their surgical management proved to be safe and led to satisfactory initial correction, it showed a high recurrence of deformity [20]. In the study conducted by Ryabykh et al., the preoperative Cobb angle for kyphosis was 83.7° (median 75°, range 45°–134°) and the mean kyphosis correction angle was 57° (median 56°, range 19°–96°) [21]. Akbar et al. conducted Gibbus surgery and postoperative rehabilitation. The mean preoperative lumbar kyphosis angle was 131° (90°–170°), and the mean postoperative kyphosis correction angle was 42° (15°–90°) [47].

##### Lumbar lordosis

In the study of Ryabykh et al., only four patients needed lordosis correction [21]. The mean preoperative Cobb angle for lordosis was 67 (median 71°, range 43°–84°) and the mean postoperative Cobb angle correction was 25° (median 28°; range 9°–35°) [21]. Crawford et al. reported five patients with an average postoperative lordosis of 34° (range 9–52°) after the initial correction, two of which retained the lordosis (5° and 27°) during the long-term follow-up [20].

#### Hip

Two studies attempted to manage hip instabilities surgically [14, 26]. Erol et al. conducted open reduction, pelvic osteotomy, proximal femoral osteotomy, and release for hip flexion contracture on 26 MMC patients with hip instabilities [26]. Of the 16 patients with unilateral dislocation and functional problems, 14 experienced improvements in gait patterns, while the other did not show any improvement [26]. Yildirim et al. evaluated the periarticular release of contractures and bony procedures, with or without muscle transfer in two groups [14]. According to Reimer’s radiographic index, the residual subluxation of the femoral head did not differ between the two groups. Despite the overall improvement of the pelvic obliquity, these two groups did not demonstrate any significant difference. The postoperative assessment showed that functional ambulatory remained similar to the preoperative status [26].

### Multidisciplinary management

Sixteen studies [9–12, 20, 21, 43–45, 49–51, 53, 55–57] had used managements other than orthopedic, including urologic, neurosurgical, neurologic, and gastrointestinal managements.

#### Urologic management

Wang et al. reported that compared to controls, SB patients required more urologic procedures (0.56% cases vs. 0.13% controls, OR = 4.4, *p* < 0.001] [50]. Lemelle et al. managed incontinency either with medical (191 patients) or surgical (230 patients) approaches [45]. The clean intermittent catheterization (CIC) was used by 116 (61%) patients in the medical treatment group. Surgical interventions included urinary non-continent diversion (Bricker procedure in 19 and vesicostomy in 4 patients), intestinal bladder augmentation (148 patients), bladder neck surgery (49 patients), detrusorotomy (4 patients), and neurostimulation (6 patients).

In a single institute analysis, Januschek et al. reported that CIC was the most common management for damaged bladder [53]. However, they had a case of uro-genito-ano-rectal malformation, which required colostomy and suprapubic vesical fistula. Despite releasing untethered cord procedures, Altiok et al. reported bladder control impairment [51]. According to the study by Goldstein et al., prophylactic spinal cord untethering before scoliosis correction surgeries improved the urologic function of 1% of the MMC patients, which is not promising [10]. Sileo et al. indicated postnatal surgical repairs on neonates with MMC, 10.9% of whom were diagnosed with hydronephrosis at the follow-up, and 81.8% with a neuropathic bladder [57]. To alleviate the complications, CIC (85.5%) and anticholinergic pharmacological therapies (87.3%) were used. Moreover, 89.1% of the infants had different levels of urinary incontinence. Two studies of Beuriat et al. [9, 11] utilized postoperative bladder managements on average as follows: non-autonomous natural rout CIC (21.74–7%), autonomous natural rout CIC (21.74–33%), non-autonomous Mitrofanoff CIC (10.87–7%), autonomous Mitrofanoff CIC (6.52–7%), and diapers (26.09–23%) published in 2018 and 2019, respectively. Spazzapan and his colleagues followed the treated MMC children for a mean of 7.7 years and found that 21% of the patients had normal bladder function, 5.2% were suffering from incontinency, and 73.6% were having neurogenic bladder [56].

Moreover, they showed that urinary dysfunction and MMC level are not significantly correlated (*p* = 0.062). North et al. performed two 10-year cohorts and reported that 82% of patients in the first cohort and 94% of patients in the second cohort benefited from CIC to manage incontinency [55]. In the study from Ulsenheimer et al., seven patients required CIC based on the vesico-urethral reflux or significant vesicle residues [44]. Anticholinergic medications was needed for seven patients. They also prescribed a combination of doxozym with oxybutynin for one patient and a combination of imypramine and oxybutynin for another one. Repeated urinary tract infections (UTI) episodes were reported in 10 patients. Also, they prescribed prophylactic antibiotics for all patients with neurogenic bladder [44].

#### Neurosurgical and neurological management

The follow-up assessments of kyphosis corrective surgery in the study of Ryabykh et al. showed improvement of modified Japanese Orthopedic Association (mJOA) neurologic scale by 0.6 points and the functional independence measure (FIM score) by 6.6 points, which represents average functional status [21]. None of the patients suffered from back pain (visual analog scale = 0). The requirement of ventriculoperitoneal (VP) shunt revision ranged from 30.5 to 86% in different studies [30, 39, 42, 44]. In the study conducted by Ozerdemoglu et al., 16 out of the 26 MMC patients required a spinal fusion ultimately [43].

#### Gastrointestinal management

Bowel management in patients suffering from MMC consists of two main categories; enema and diapers. Two studies by Beuriat et al. [9, 11] reported the rate of using enema as (34.78–24%) and diapers as (30.43–21%) published in 2018 and 2019, respectively. Applying suppositories, cecostomy tubes, digital sweeps, oral medication, diet control, and antegrade colonic enema (ACE) are other proposed management [55, 57]. Spazzapan et al. used colostomy in 5.2% of patients to manage bowel incontinency [56].

## Discussion

This review is the first to summarize outcomes and applications of various multidisciplinary orthopedic, urologic, neurologic, and gastrointestinal management of patients with MMC with an orthopedic focus. The orthopedic management of MMC tends to be based on musculoskeletal problems. MMC patients encounter congenital and acquired deformities, which can affect their quality of life. Congenital deformities include scoliosis, kyphosis, teratological hip dislocations, club feet, and flat feet with vertical talus.

Collectively, for MMC patients with mild to moderate scoliosis (a flexible curve < 50°), non-operative treatment is suggested (e.g., sitting supports, spinal orthoses, and functional strengthening programs) to help them improve their independent functions [22, 61]. However, for MMC patients with severe scoliosis, the treatment plan is based on whether they are ambulatory or not. Orthopedists mainly offer surgical treatment for non-ambulatory patients who suffer from sitting or skin deformities due to spine curvature [25, 62]. Regarding ambulatory patients, surgeons must decide case by case and consider the pros and cons of surgical intervention, mainly spinal fusion [25, 62, 63]. Possible complications are implant problems [64] (e.g., implant failure, dislocation, and pseudoarthrosis [25]) and wound infections [25].

Considering hip deformities, maintaining hip range of motion should be the orthopedist’s treatment goal. Formerly, most hip surgical interventions were performed to reduce paralytic hip dislocations, but they were not successful in achieving the ability to ambulate, lessening the need for bracing, or relieving pain [27–29, 65]. In contrast, currently, hip surgeries aim to release contractures. It is worth mentioning that treatment plan varies based on the level of myelomeningocele, whether sacral-level or thoracic and lumbar-level. Ultimately, surgeons should consider that maintaining a level pelvis and free motion of the hips rather than radiographic reduction of the hip are primary goals of the treatment plan [28]. Additionally, loss of motion and pathologic fractures are among surgical complications hindering ambulation [28, 29].

As torsional abnormalities of the lower extremities often occur in MMC patients, it is essential to manage them if it interferes with their gait and ambulation. For non-ambulatory patients, orthopedic surgeon aims to minimize bracing requirements while achieving as normal a gait as possible. It is generally suggested to delay the surgery until the patient is 6 years old to decrease the risk of recurrence, and till then, orthotic management is helpful to maintain gait function [66]. Rotational osteotomy is the foremost choice of surgery in case of severity and difficulty with orthoses [67].

Regarding foot and ankle issues management, the orthopedist’s treatment goal includes a plantigrade, flexible, braceable foot on which a shoe can be worn to allow ambulation. Conservative management with passive manipulation is mainly offered for MMC patients with equinus, calcaneus, and mild positional clubfoot. For instance, severe clubfoot deformities may require surgical correction following the radical posteromedial-lateral release (PMLR) method, which leads to controversial outcomes due to the motor level of involvement [68]. Calcaneous feet treatment was reported following the anterior or anterolateral soft-tissue release with an 82% success rate [69]. Distal tibia valgus deformity could be corrected by medial hemiepiphysiodesis of the distal tibia or, in severe cases, distal tibia osteotomy. For hindfoot valgus, treatment consists of a medial displacement osteotomy of the calcaneus [18].

In children with MMC, orthoses are used to maintain alignment, prevent deformity, correct flexible deformities, facilitate independent mobility, and protect the insensate limb. Orthoses are used for upright weight-bearing and mobility in thoracic and lumbar MMC patients. Due to absent hip extensors and abductors and ankle plantar- and dorsi-flexors, orthoses could help MMC patients with low lumbar and sacral level involvement s[70].

The majority of the included studies performed postnatal management and operations rather than prenatal. Although controversies exist between surgeons, postnatal repair is established as a worldwide standard for MMC patients [71]. Concurrently, the MOMS (the management of myelomeningocele study) has indicated that prenatal repair increased maternal and fetal complication risks. The outcomes were significantly better than postnatal repair in decreasing the need for VP shunt and hydrocephalus occurrence (40% vs. 82%), as well as functional improvement. The MOMS cohort assessed gait independence at the age of 30 months. As a result, more of the prenatal group had independent gait versus postnatal (40% vs. 21%) [72]. Prenatal repair of MMC is categorized as the neurosurgical management of MMC. We should consider the delivery method (cesarean section or vaginal delivery) to secure the neonate, neural tissues, and MMC sac [71]. However, Greene et al. reported no significant difference between the mode of delivery (cesarean and vaginal delivery) and patients’ motor functions [73]. Regarding urological complications, MMC patients undergoing fetal (prenatal) surgery require neurogenic bladder management, similar to patients experiencing postnatal surgeries [74].

Ambulation status is one of the features taken into account for assessing the efficacy and effectiveness of the conducted procedures. The ambulation status is influenced by spasticity, contractures, syringohydromyelia, and musculoskeletal problems including, foot, knee, and hip deformities and scoliosis. Moreover, it has been suggested that the neurologic impairment level is a significant factor for ambulation. Although shunting is widely used as prenatal management, the history of shunt insertion or revision has a negative effect on ambulation status [75–77]. In the orthopedic field, surgeons correct spinal deformities to support muscle balance and sitting stability. Also, orthopedists can release hamstrings and treat knee flexion contractures in ambulatory patients. The valgus deformity can also affect ambulation due to muscle imbalance [78]. Although utilizing assistive devices may decrease the number of steps per day, it is a positive factor for predicting walking activity in MMC [79].

Shreds of evidence show that untethering the spinal cord prior to scoliosis surgery may be redundant in patients who do not characterize symptoms (e.g., pain, weakness, gait abnormality, lower extremity, hip and foot deformity, urological changes, and rapidly progressive scoliosis) [80].

Pediatric urologists’ experience confirms that clean intermittent catheterization (CIC) may lessen renal deterioration risk. Moreover, in some cases, CIC might be influential on renal function improvement. The success rate of the CIC program hinges on several factors (e.g., physiological, developmental, motivational) [81].

The ultimate aim of management in MMC patients is to improve their quality of life. To assess the quality of life, there is a controversy regarding the person who should take the survey: the patients, their parents, or both. Studies measuring patients’ health status with MMC have reported a lower health-related quality of life (HRQOL) than the general population [82–85]. On the other hand, exercise and fitness showed an essential impact on higher HRQOL [86]. Some studies have introduced predictors for HRQOL, including the need for support and an assistant to supervise them. In contrast, lesion level and social status had no significant effect on overall HRQOL [82]. Besides, managements like bladder augmentation increased patients’ HRQOL by reducing the need for catheterization [87].

Despite all efforts for the management of MMC, it should be noted that lesser the time we waste, the better outcome we can achieve. Therefore, more equipped multidisciplinary centers should be established to give MMC patients the care and management they need and help them acquire a better quality of life. This approach brings together patients and specialists experienced in different disciplines. Moreover, the modern world has introduced robotic-assisted surgeries in the medical community, which could help surgeons accomplish better outcomes and reduce surgical complications. It is also suggested to conduct more studies on prenatal repair surgeries to compare their application, outcomes, and complications with postnatal repair surgeries.

This study encountered several limitations. We did not comprehensively review managements other than orthopedics due to the specific focus of our study. Included studies were mainly retrospective analyses rather than RCTs, which are critically needed to achieve more robust results. Another limitation of this study was that included studies did not have a homogeneous size of samples and surgery techniques. Further multivariate analyses are required to reveal each management’s effects on MMC patients’ HRQOL.

## Conclusion

Timely and proper multidisciplinary management is critical for MMC given its high burden and psychosocial consequences. Orthopedic approaches play a key role in MMC management by alleviating spinal deformities, particularly scoliosis, and hip, foot, and ankle complications. However, the most appropriate management, whether surgical or non-surgical, may vary for different patients, given disease severity and the age of patients.

## Data Availability

Not applicable

## Abbreviations

ACE: Antegrade colonic enema
CIC: Clean intermittent catheterization
EUROCAT: European Surveillance of Congenital Anomalies
FIM: Functional independence measure
HRQOL: Health-related quality of life
mJOA: Modified Japanese Orthopedic Association
MMC: Myelomeningocele
MOMS: The management of myelomeningocele study
NTD: Neural tube defect
SB: Spina bifida
TCR: Tethered cord release surgery
UTI: Urinary tract infection
VDS: Ventral derotational spondylodesis
VP: Ventriculoperitoneal
